# Randomized Phase 3 Trial Evaluating the Safety, Tolerability, and Immunogenicity of V114, a 15-Valent PCV, Followed by PPSV23 6 Months Later (PNEU-DAY): Subgroup Analysis in Adults 18–49 Years of Age Enrolled at Center for Indigenous Health Sites

**DOI:** 10.3390/vaccines14010003

**Published:** 2025-12-19

**Authors:** Laura L. Hammitt, Ulrike K. Buchwald, Jennifer McCauley, Tulin Shekar, Wei Fu, Kyeongmi Cheon, Tina Sterling, Gretchen Tamms, Natalie Banniettis, Luwy Musey, Jason J. LeBlanc, Robert Weatherholtz, Dennie Parker Riley, Estar Denny, Carol Tso, Kristen Roessler, Mathuram Santosham

**Affiliations:** 1 Department of International Health, Center for Indigenous Health, Johns Hopkins Bloomberg School of Public Health, Baltimore, MD 21205, USA; rweathe1@jhu.edu (R.W.); dparke71@jhu.edu (D.P.R.); estar.denny01@frontier.edu (E.D.); ctso1@jhu.edu (C.T.); kroessl1@jhu.edu (K.R.); msantosham@jhu.edu (M.S.); 2 Merck & Co., Inc., Rahway, NJ 07065, USA; ulrike.buchwald@merck.com (U.K.B.); jennifer.mccauley@merck.com (J.M.); tulin.shekar@merck.com (T.S.); wei.fu3@merck.com (W.F.); kyeongmi.cheon@merck.com (K.C.); tina_sterling@merck.com (T.S.); gretchen_tamms@merck.com (G.T.); natalie.banniettis@merck.com (N.B.); museyluwy@gmail.com (L.M.); 3 Division of Microbiology, Department of Pathology and Laboratory Medicine, Nova Scotia Health, Halifax, NS B3H 1V8, Canada; jason.leblanc@nshealth.ca; 4 Department of Pathology, Faculty of Medicine, Dalhousie University, Halifax, NS B3K 6R8, Canada

**Keywords:** pneumococcal vaccine, V114, 15-valent PCV, PCV13, PPSV23, American Indian

## Abstract

Background/Objectives: American Indian/Alaska Native individuals exhibit a higher prevalence of carriage of *Streptococcus pneumoniae* and are at increased risk of invasive pneumococcal disease compared with the general US population, driven by persistent inequities in health determinants. Although the use of pneumococcal vaccines has reduced carriage of vaccine serotypes, the prevalence of carriage of non-vaccine serotypes has increased. Methods: This study was a descriptive subgroup analysis of the PNEU-DAY study (NCT03547167; EudraCT 2017-004915-38). Safety, tolerability, and immunogenicity of sequential administration of either V114, a 15-valent pneumococcal conjugate vaccine (PCV), or 13-valent PCV (PCV13), followed 6 months later by 23-valent pneumococcal polysaccharide vaccine (PPSV23), were evaluated in pneumococcal vaccine-naïve American Indian adults with or without pre-defined risk factors for pneumococcal disease. Polymerase chain reaction testing assessed nasopharyngeal/oropharyngeal carriage of *S. pneumoniae*. Results: Following administration of PCV and PPSV23, the proportions of participants with adverse events were generally comparable between vaccination groups. V114 and PCV13 were immunogenic for all respective vaccine serotypes, with V114 inducing robust immune responses to the two additional serotypes not included in PCV13 (22F and 33F), based on opsonophagocytic activity geometric mean titers and immunoglobulin G geometric mean concentrations at 30 days post-vaccination. Sequential administration with PPSV23 was immunogenic in both vaccination groups. Nasopharyngeal/oropharyngeal carriage of *S. pneumoniae* was observed in 16.7% to 22.6% of American Indian participants across the study timepoints. Conclusions: V114 was well tolerated and immunogenic for the 15 serotypes in V114 when administered either alone or followed by PPSV23. Use of V114 has the potential to expand serotype coverage and protect against pneumococcal disease resulting from serotypes absent in PCV13 among American Indian adults.

## 1. Introduction

Pneumococcal disease (PD) caused by *Streptococcus pneumoniae* is a significant cause of morbidity, hospitalization, and mortality across all ages [1,2,3]. American Indian/Alaska Native individuals, who comprise approximately 2.9% of the total population of the United States [4], have a higher prevalence of nasopharyngeal (NP) carriage of *S. pneumoniae* and experience a three-to-five-fold-higher risk of invasive PD (IPD) when compared with the overall population of the United States [5,6,7,8]. Disparities in socioeconomic conditions and access to healthcare, in addition to a high prevalence of chronic comorbidities, including diabetes mellitus, chronic heart disease, and asthma, contribute to the elevated burden of PD in American Indian/Alaska Native communities [1,6,9,10,11,12,13].

In the United States, two types of vaccine are recommended for pneumococcal immunization: pneumococcal conjugate vaccines (PCVs) and a 23-valent pneumococcal polysaccharide vaccine (PPSV23) [14,15]. PPSV23 is a vaccine that has been recommended since the 1980s for older adults and individuals with certain underlying conditions that increase the risk of PD [14,16,17,18]. Until recently, 13-valent PCV (PCV13), which contains 13 serotypes, was recommended by the Advisory Committee on Immunization Practices (ACIP) in adults ≥65 years of age with or without chronic medical conditions based on shared clinical decision-making [19,20]; for adults ≥19 years of age with immunocompromising conditions, vaccination with PCV13 followed by PPSV23 was recommended [19]. While the use of pneumococcal vaccines among the American Indian/Alaska Native population has led to a substantial decrease in IPD caused by vaccine serotypes and vaccine-type carriage prevalence [5,21], the prevalence of non-vaccine serotype carriage has increased [21], highlighting the need for new PCVs with broader serotype coverage.

Three higher-valency PCVs were recently approved for use in adult populations [22,23,24]. V114 (VAXNEUVANCE™, Merck Sharp & Dohme LLC, a subsidiary of Merck & Co., Inc., Rahway, NJ, USA [MSD]) is a 15-valent PCV that contains the 13 serotypes in PCV13 plus two epidemiologically important serotypes (22F and 33F) that cause IPD [20,25,26,27,28,29,30,31]. V114 is approved for use in the United States and globally for the prevention of PD in adults ≥18 years of age [25,32,33]. V116 (CAPVAXIVE™, MSD) is an adult-specific, 21-valent PCV containing the serotypes most often associated with IPD in adults from regions with established pediatric PCV programs; V116 is approved in multiple regions, including the United States, Canada, Australia, the European Union, and Japan, among others, for the prevention of IPD in individuals ≥18 years of age, and for the prevention of pneumonia in individuals ≥18 years of age in the United States and the European Union [22,34,35,36,37,38]. A 20-valent PCV (PCV20), which contains 20 serotypes, was also recently approved in adults ≥18 years of age [33,39]. In October 2024, the ACIP revised and expanded the adult pneumococcal immunization recommendations: for individuals ≥19 years of age with particular risk factors for PD and for all adults ≥50 years of age, vaccination is recommended with either a single dose of V116 or PCV20 or V114 followed by PPSV23 ≥1 year later [40,41].

PNEU-DAY was a descriptive Phase 3 study assessing the safety, tolerability, and immunogenicity of V114 or PCV13 administered on Day 1, as well as PPSV23 6 months later, among 1515 immunocompetent pneumococcal vaccine-naïve adults 18–49 years of age with or without pre-defined risk factors for PD [42]. In this descriptive subgroup analysis of PNEU-DAY, we evaluated the safety, tolerability, and immunogenicity of V114 in American Indian adults as compared with PCV13. In addition, NP/oropharyngeal (NP/OP) carriage of *S. pneumoniae* was assessed in this population.

## 2. Materials and Methods

### 2.1. Study Design and Participants

In the primary study conducted between July 2018 and July 2020 (Protocol V114-017; PNEU-DAY study; registered with Clinicaltrials.gov: NCT03547167 [trial registration date: 24 May 2018] and the European Union Clinical Trials Register: EudraCT 2017-004915-38 [trial registration date: 4 October 2018]; the full protocol and statistical analysis plan for the primary study are available online [43]), approximately 600 American Indian participants 18–49 years of age with and without risk factors for PD were recruited between October 2018 and June 2019 and enrolled at five sites of the Johns Hopkins Center for Indigenous Health (CIH; formerly the Center for American Indian Health) located in the southwest region of the United States (a list of sites and investigators is provided in Table S1). This descriptive subgroup analysis was conducted to evaluate the safety, tolerability, and immunogenicity of V114 compared with PCV13 in this population, and to describe pneumococcal NP/OP carriage. Participants in this subgroup analysis met eligibility criteria for the PNEU-DAY study (Table S2) [43]. In brief, participants were eligible if they had not previously received a pneumococcal vaccine and were American Indian adults in good health or with any of the following risk conditions for PD: chronic heart disease, chronic liver disease, chronic lung disease, diabetes mellitus, and tobacco use. The sample size was selected to reach a sufficient number of participants across different age groups and risk factor categories with exposure to V114 [42].

Participants were randomized 3:1 to receive a single dose of either V114 or PCV13 on Day 1, and a subsequent single dose of PPSV23 administered at Month 6. Randomization was implemented using an interactive response technology system and was stratified based on enrollment site and type/number of pre-defined baseline risk factors [42,43]. The participants and investigators involved in clinical evaluation remained blinded to the group assignments. In addition, participants were all screened for alcohol misuse/abuse using the Alcohol Use Disorder Identification Test Alcohol-Consumption (AUDIT-C) test; potentially harmful alcohol use (scores of ≥5) counted as an additional risk factor for stratification purposes [42,44].

### 2.2. Vaccines and Administration

V114 (VAXNEUVANCE™, MSD) contains pneumococcal capsular polysaccharides from serotypes 1, 3, 4, 5, 6A, 6B, 7F, 9V, 14, 18C, 19A, 19F, 23F, 22F, and 33F [25,42].

PCV13 (Prevnar 13^©^, Wyeth LLC, marketed by Pfizer, New York, NY, USA) contains pneumococcal capsular polysaccharides from serotypes 1, 3, 4, 5, 6A, 6B, 7F, 9V, 14, 18C, 19A, 19F, and 23F [20,42].

PPSV23 (PNEUMOVAX^®^ 23, MSD) contains pneumococcal capsular polysaccharides from serotypes 1, 2, 3, 4, 5, 6B, 7F, 8, 9N, 9V, 10A, 11A, 12F, 14, 15B, 17F, 18C, 19A, 19F, 20, 22F, 23F, and 33F [16,42].

The three vaccines were supplied, stored, and administered per a previous report [42]. A 0.5 mL dose of V114 (lot number 00068290) or PCV13 (lot numbers 0000793304, 0000921112, and 0000814723) and PPSV23 (lot number 0000794346) were administered by study personnel who were unblinded and not otherwise involved in the conduct of the study.

### 2.3. Study Assessments and Analyses

#### 2.3.1. Safety and Immunogenicity

Adverse events (AEs) occurring after each study vaccine were ascertained, including any unsolicited non-serious AEs within 14 days of vaccination and any serious AEs (SAEs) occurring from Day 1 through Month 7. Solicited injection-site events were collected from Days 1–5 following vaccination and were designated as vaccine-related. Solicited systemic events were collected from Days 1–14 following vaccination; vaccine-relatedness was determined by the investigators. Safety analyses for the subset of participants enrolled at CIH sites were conducted on the all-participants-as-treated population (i.e., participants who received the study vaccine for the timepoint of interest) and were reported as proportions.

To assess immune responses, blood samples were drawn pre-PCV vaccination (Day 1), 30 days post-PCV vaccination (Day 30), pre-PPSV23 vaccination (Month 6), and 30 days post-PPSV23 vaccination (Month 7), as previously described [42]. Serotype-specific opsonophagocytic activity (OPA) geometric mean titers (GMTs) and serotype-specific immunoglobulin G (IgG) geometric mean concentrations (GMCs) were assessed in the subset of participants enrolled at CIH sites who were among the per-protocol population, which included all randomized participants with no protocol deviations that could affect the immunogenicity results in a substantial way.

Safety and immunogenicity of study PCV followed by PPSV23 were also analyzed in subgroups based on age (18–29, 30–39, and 40–49 years of age) and number of baseline risk factors for PD (0 or ≥1 risk factors).

#### 2.3.2. NP/OP Carriage of Vaccine-Preventable Serotypes

Approximately 300 American Indian participants who were part of the CIH cohort of the primary study were further included in a substudy to evaluate the relationship between NP/OP carriage of *S. pneumoniae* and pneumococcal vaccination on the performance of a serotype-specific urinary antigen detection (SSUAD) assay. The methodology and characteristics of the SSUAD assay have been included in a different manuscript; in this current analysis, overall pneumococcal NP/OP carriage data in this population and carriage of vaccine serotypes are further analyzed.

#### 2.3.3. Detection and Serotyping of *S. pneumoniae* from NP/OP Analyses

NP/OP flocked swabs were collected immediately prior, to and up to, 30 days following each vaccination (on Days 3, 8, 15, and 30 following V114/PCV13; on Days 183, 188, 195, and Month 7 following PPSV23), using standard procedures [45,46]. Total nucleic acids were purified from NP/OP swab material, and detection and serotyping of *S. pneumoniae* in NP/OP swabs were performed using previously described methods [46,47,48]. In brief, sequential real-time polymerase chain reaction (PCR) was used to target autolysin (*lytA*), capsular polysaccharide synthesis gene A (*cpsA*), and the pneumococcal iron acquisition (*piaA*) gene. *S. pneumoniae* carriage positivity was determined if *lytA* PCR was positive, along with a positive result from *cpsA* or *piaA*; only these specimens were then subjected to triplex real-time multiplex PCRs spanning the 15 serotypes covered in V114 [46].

## 3. Results

### 3.1. Study Population

**Figure 1:**
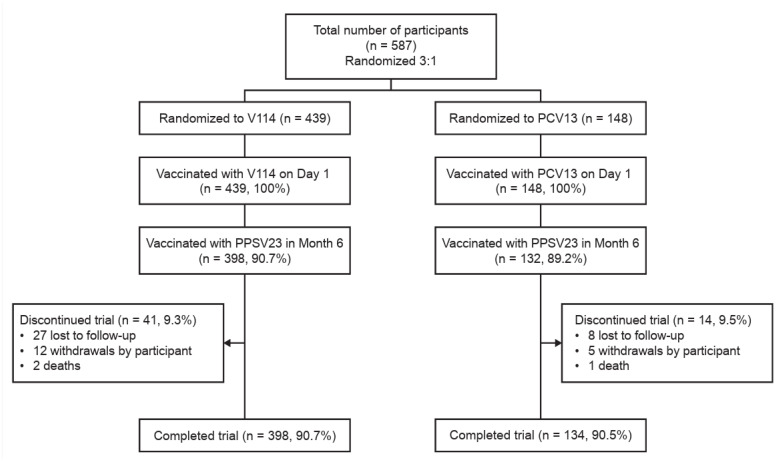
Participant flow chart. Data are for participants enrolled at Center for Indigenous Health sites. The denominator for calculations is based on the number randomized. The “completed trial” classification may include participants who missed PPSV23 administration. Abbreviations: PCV13, 13-valent pneumococcal conjugate vaccine; PPSV23, 23-valent pneumococcal polysaccharide vaccine; V114, 15-valent pneumococcal conjugate vaccine.

Between October 2018 and July 2020, a total of 587 randomized participants from CIH sites were included in this analysis (Figure 1). All randomized participants were vaccinated with V114 or PCV13 in a 3:1 ratio, with 439 receiving V114 and 148 receiving PCV13. Nearly all participants (90.3%) were vaccinated with PPSV23 in Month 6. Most participants (90.6%) completed the study. All intervention groups had similar discontinuation rates.

Across both vaccination groups, participant characteristics were generally comparable at baseline (Table 1). The mean age of the study participants was 32.6 years, and about 58% of participants were female. Approximately 65% of enrollees did not have protocol-defined risk factors for PD at screening.

### 3.2. Safety

#### 3.2.1. Following Vaccination with PCV (Days 1–30)

**Figure 2:**
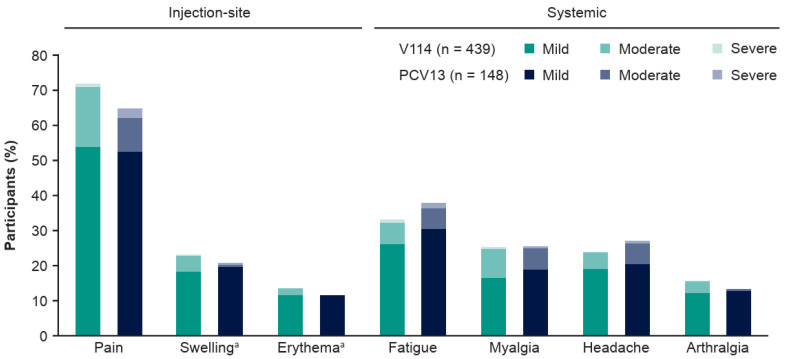
Post-vaccination solicited AEs, by study group. Data are for participants enrolled at Center for Indigenous Health sites. Injection-site events were solicited from Days 1–5 following vaccination. Systemic events were solicited from Days 1–14 following vaccination. ^a^ For solicited injection-site swelling and injection-site erythema, mild events measured >0–≤5 cm, moderate events measured >5–≤10 cm, and severe events measured >10 cm. Abbreviations: AE, adverse event; PCV13, 13-valent pneumococcal conjugate vaccine; V114, 15-valent pneumococcal conjugate vaccine.

Following administration of PCV, the proportions of participants with AEs, including solicited AEs, were generally comparable between the V114 and PCV13 groups (Table 2). No vaccine-related SAEs were reported in either group. Three deaths were reported across both vaccination groups (two in the V114 group and one in the PCV13 group), none of which were considered by the investigator to be related to the study vaccine. The most common solicited AE was injection-site pain (V114: 72.0%; PCV13: 64.9%). Fatigue was the most frequently reported solicited systemic AE in both groups (V114: 33.3%; PCV13: 37.8%). In both vaccination groups, most solicited AEs were mild (Figure 2; Table S3).

In a subgroup analysis by age (18–29, 30–39, and 40–49 years), V114 was well tolerated within each age group (Table S4). Following administration of V114, solicited AEs were observed in 79.3%, 73.3%, and 80.8% of participants in the 18–29, 30–39, and 40–49 years of age groups, respectively. The proportions of participants with solicited AEs within each age group were generally comparable between the V114 and PCV13 groups.

V114 was well tolerated, regardless of whether risk factors for PD (0 or ≥1) were present at baseline. The proportions of participants experiencing solicited AEs were generally comparable between participants with or without risk factors for PD (Table S5).

#### 3.2.2. Following Vaccination with PPSV23 (Months 6–7)

Following administration of PPSV23, the majority of participants in both vaccine groups experienced at least one AE (Table 2). As with the Day 1 vaccination, pain was the most frequently reported solicited injection-site AE (V114: 55.0%; PCV13: 56.1%) and fatigue was the most frequently reported solicited systemic AE (V114: 24.9%; PCV13: 23.5%). The majority of solicited AEs were mild (Figure S1; Table S3).

### 3.3. Immunogenicity

#### 3.3.1. Following Vaccination with PCV (Days 1–30)

**Figure 3:**
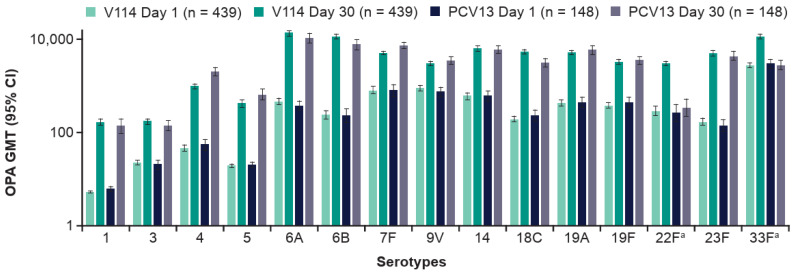
Serotype-specific OPA GMTs by timepoint and study group. Data are for participants enrolled at Center for Indigenous Health sites. The within-group 95% CIs were obtained by exponentiating the CIs of the mean of the natural log values based on the t-distribution. ^a^ Additional serotypes in V114. Abbreviations: CI, confidence interval; GMT, geometric mean titer (1/dilution); OPA, opsonophagocytic activity; PCV13, 13-valent pneumococcal conjugate vaccine; V114, 15-valent pneumococcal conjugate vaccine.

V114 induced immune responses for all 15 V114 serotypes at 30 days post-vaccination, as assessed by OPA GMTs (Figure 3; Table S6) and IgG GMCs (Table S7). PCV13 was immunogenic for all 13 PCV13 serotypes at 30 days post-vaccination.

**Figure 4:**
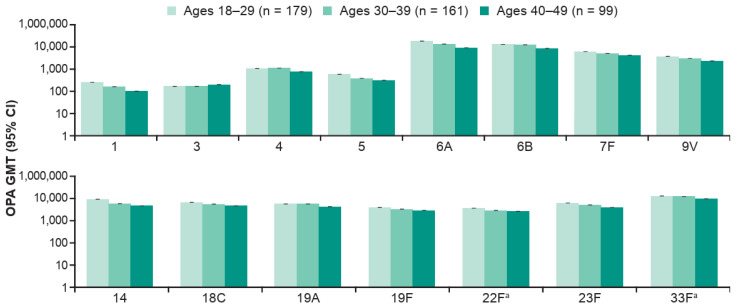
Serotype-specific OPA GMTs at 30 days post-vaccination (Day 30) with V114, by age. Data are for participants enrolled at Center for Indigenous Health sites. The within-group 95% CIs were obtained by exponentiating the CIs of the mean of the natural log values based on the t-distribution. ^a^ Additional serotypes in V114. Abbreviations: CI, confidence interval; GMT, geometric mean titer (1/dilution); OPA, opsonophagocytic activity; V114, 15-valent pneumococcal conjugate vaccine.

V114 induced immune responses for all 15 V114 serotypes, regardless of participant age or presence of risk factors for PD, as assessed by OPA GMTs at 30 days post-vaccination with PCV (Tables S8–S11). There was a suggestion of lower serotype-specific OPA GMTs in those 40–49 years of age compared with those 18–29 years of age (Figure 4). OPA GMTs were generally comparable between the vaccination groups for most of the shared serotypes within each age group and whether or not risk factors for PD were present.

#### 3.3.2. Following Vaccination with PPSV23 (Months 6–7)

**Figure 5:**
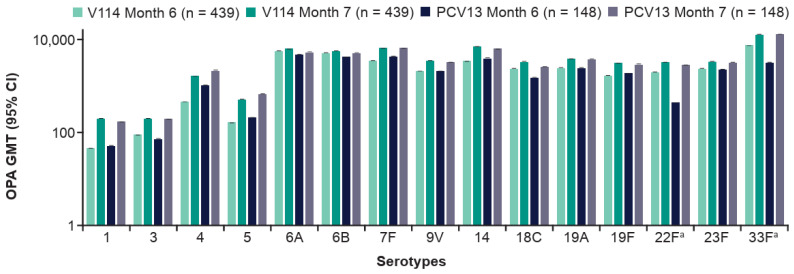
Serotype-specific OPA GMTs before and 30 days after PPSV23 vaccination. Data are for participants enrolled at Center for Indigenous Health sites. The within-group 95% CIs were obtained by exponentiating the CIs of the mean of the natural log values based on the t-distribution. ^a^ Additional serotypes in V114. Abbreviations: CI, confidence interval; GMT, geometric mean titer (1/dilution); OPA, opsonophagocytic activity; PCV13, 13-valent pneumococcal conjugate vaccine; PPSV23, 23-valent pneumococcal polysaccharide vaccine; V114, 15-valent pneumococcal conjugate vaccine.

Vaccination with PPSV23 at 6 months after the V114 or PCV13 PCV vaccination induced immune responses. At 30 days after the PPSV23 vaccination (i.e., at Month 7), serotype-specific OPA GMTs (Figure 5; Table S6) and IgG GMCs (Table S7) in both vaccination groups were generally comparable for all 15 serotypes, including serotypes 22F and 33F. A consistent booster response was not observed following vaccination with PPSV23 across the serotypes shared between V114/PCV13 and PPSV23 (Figure 5).

### 3.4. NP/OP Carriage Results

The proportion of participants who were PCR-positive for pneumococcal NP/OP carriage at a given visit ranged from 16.7% to 22.6% (Table 3). Small differences in pneumococcal carriage were seen between the V114 and PCV13 vaccination groups; however, given the 3:1 randomization, these findings are to be interpreted with caution. The NP/OP carriage prevalence differed by timepoint and ranged between 17.3% at Day 183 and 22.6% at Day 1 in the combined vaccination groups. The serotype-specific NP/OP carriage prevalence of specific serotypes varied by serotype and over time (Table S12). Serotype-specific carriage prevalences for serotypes 1, 4, 5, 9V, and 33F were approximately 4–7% on Day 1. The highest carriage detection rates for the two additional serotypes in V114 in the total cohort were 2.7% at Day 1 for serotype 22F and 6.0% at Day 1 for serotype 33F; group-specific differences in colonization rates for these two serotypes should be interpreted with caution, given small sample numbers. Colonization with other vaccine serotypes of *S. pneumoniae* was very low or not detected.

## 4. Discussion

This subgroup analysis demonstrated that V114 followed by PPSV23 was immunogenic and generally well tolerated in American Indian adults 18–49 years of age. These results are consistent with those observed in the overall study population [42].

In this substudy, administration of V114 or PCV13 followed by PPSV23 was well tolerated by participants. No vaccine-related SAEs were reported in either group. Solicited events accounted for most of the AEs and vaccine-related AEs; the majority of solicited AEs were mild to moderate in severity. When stratified based on age and the number of risk factors for PD, V114 was well tolerated within each age and risk factor group. While a relatively lower percentage of participants experienced solicited injection-site AEs with PCV13 when compared with V114, it is not possible to make formal comparisons between the vaccination groups, as this study was not designed to evaluate differences between the V114 and PCV13 vaccination groups. Statistical comparisons between V114 and PCV13 in a pivotal Phase 3 study in adults found that solicited systemic AEs were comparable between groups and the majority were reported as mild [49].

V114 induced immune responses against the two additional serotypes in V114, 22F and 33F, which are among the most common serotypes that cause IPD globally and were collectively responsible for 6.3% and 0.9% of IPD cases during 2011–2019 among Navajo children and adults, respectively [26,27,50,51,52,53,54,55,56]. In addition, immune responses to the 13 serotypes in common for the two vaccines were generally comparable among recipients of PCV13 and V114. This is further supported by the results of a pivotal Phase 3 study comparing V114 with PCV13, in which V114 was non-inferior for the 13 shared serotypes and was superior for the two unique serotypes 22F and 33F, as well as for the shared serotype 3 [49]. Taken together, these results suggest that V114 has the potential to expand protection to these two additional serotypes while maintaining protection against the serotypes that V114 has in common with PCV13.

PPSV23 administered 6 months following V114 vaccination induced immune responses to all 15 serotypes included in V114; these immune responses were generally similar to those elicited with PPSV23 administered 6 months following PCV13 vaccination. Following vaccination with PPSV23, a consistent booster response was not observed for certain serotypes in the V114 group and the PCV13 group; however, this is unlikely to be of clinical significance, given that post-PPSV23 antibody GMCs and GMTs were high. Immunogenicity findings were similar between vaccination groups for serotype 3, as well as other serotypes, such as 33F. Results from the primary study reported that, in general for some shared serotypes, OPA and IgG responses following receipt of PPSV23 were lower than those measured post-PCV [42]. Taken together, these results suggest that the 6-month interval between administration of PCV and PPSV23 may not have been sufficient to generate a robust booster response to serotypes common between them. Similar findings were reported in a study among adults 60–64 years of age that evaluated PCV13 followed 1 year later by PPSV23 [57]. OPA and antibody responses to the serotypes that PCVs share with PPSV23 may be greater with a longer interval between vaccines [58].

The benefits of sequential vaccination with PCV followed by PPSV23 due to the broadening of serotype coverage have been reported with PCV13 [59,60,61]. A study evaluating PCV13 alone compared with PCV13 followed by PPSV23 2 months later demonstrated superiority of immune responses against serotypes unique to PPSV23 and non-inferiority of shared serotypes at 3 months post-PCV administration [59]. As V114 has demonstrated immunogenicity to the unique serotypes 22F and 33F [49], sequential vaccination using V114 plus PPSV23 could potentially further expand serotype coverage over PCV13 plus PPSV23 [62,63]. Additional studies on the correlates of protection, as well as head-to-head evaluations of differing interval lengths, would be helpful to assess the potential benefit of sequential vaccination with different time intervals [64].

V114 was immunogenic across age ranges, with a suggestion of lower immune responses in older age groups, which is consistent with previous results of PCVs in older adults and may be attributed to immunosenescence [65,66]. The presence of chronic medical conditions did not affect the immunogenicity of V114, as immune responses were generally similar between participants with or without risk factors.

This study also measured *S. pneumoniae* NP/OP carriage among American Indian participants. While the primary purpose of the NP/OP substudy was to assess any associations between pneumococcal carriage and/or pneumococcal vaccination and performance of a pneumococcal SSUAD assay (results to be published separately), this also afforded the opportunity to characterize NP/OP carriage in this population at increased risk of PD. Pneumococcal carriage is a prerequisite for PD and has been shown to be correlated with the transmission of *S. pneumoniae* in several studies [67,68]. Both NP and OP samples were analyzed to increase sensitivity [69]. The proportion of participants in the total population who were positive for pneumococcal NP/OP carriage at a given visit ranged from 16.7% to 22.6%. The carriage detected in this study is similar to that detected in previous studies among American Indian adults; however, differences in laboratory methods used to detect pneumococci preclude direct comparison. In 2010–2012, the prevalence of pneumococcal NP carriage among American Indian adults in the southwest United States was 12% using culture-based methods [7]. In 2015–2017, NP/OP pneumococcal carriage among American Indian adults in the southwest United States was 8% by culture; by PCR, NP carriage was 9.2% while OP carriage was 22.1% [70]. In this study, PCR detection of *S. pneumoniae* carriage in swabs varied across timepoints. The real-time PCR assays used in this study were qualitative and, therefore, did not allow quantitative assessments of density following vaccination with PCVs.

Of note, real-time PCR targeting *lytA* or serotype-specific genes alone on OP samples may give false-positive results due to the diversity of streptococci found in the oropharynx [47,71,72,73,74]. To reduce the potential for false-positive results, only samples with a positive PCR on two pneumococcal targets (i.e., *lytA*, *cpsA*, or *piaA*) underwent serotype-specific PCR testing; this approach may have missed the detection of vaccine serotype carriage for some individuals.

Serotypes 1, 4, 5, 9V, and 33F were the most common serotypes found in NP/OP samples. Of note, the prevalence of serotype 4 carriage was higher than reported in previous studies in this setting (0% by NP culture in 2010–2012, 1.6% by NP/OP PCR in 2015–2017, and 5.3–6.0% by NP/OP PCR across the study visits reported herein) [70]. This apparent increase in the carriage of serotype 4 among adults presaged an increase in serotype 4 IPD that has recently been reported in the Navajo Nation [75]. The two additional serotypes included in V114 but not in PCV13 (i.e., serotypes 22F and 33F) were carried by up to 10% of participants. These two serotypes were relatively less common in a similar carriage study carried out at CIH sites between 2015 and 2017 (33F in 1.8% and 22F in 0.7% of adult samples) [70]. However, it is uncertain whether these differences were real or potentially caused by differences in testing methods.

This analysis of safety and immunogenicity in American Indian adults was descriptive, thereby limiting direct comparison between the vaccination groups. The comparisons provided here refer to observed trends in immune responses but do not suggest clinically meaningful differences between the V114 and PCV13 vaccination groups. In addition, non-vaccine serotypes were not evaluated in this analysis.

## 5. Conclusions

In American Indian adults 18–49 years of age, V114 administered alone or sequentially with PPSV23 had an acceptable reactogenicity and safety profile and induced immune responses to all V114 serotypes. Among American Indian adults, who are at increased risk of PD, V114 may extend protection against IPD to two clinically important serotypes not included in PCV13.

## Figures and Tables

**Table 1:** Baseline Characteristics for Vaccinated Participants.

n (%)	V114	PCV13	Total
Vaccinated participants	439 (100)	148 (100)	587 (100)
**Sex**			
Female	258 (58.8)	81 (54.7)	339 (57.8)
Male	181 (41.2)	67 (45.3)	248 (42.2)
**Age, years**			
18–29	179 (40.8)	64 (43.2)	243 (41.4)
30–39	161 (36.7)	48 (32.4)	209 (35.6)
40–49	99 (22.6)	36 (24.3)	135 (23.0)
Mean	32.7	32.1	32.6
Range	19–49	18–49	18–49
**Race**			
American Indian	439 (100)	148 (100)	587 (100)
**Ethnicity**			
Not Hispanic/Latino	422 (96.1)	137 (92.6)	559 (95.2)
Hispanic/Latino	17 (3.9)	11 (7.4)	28 (4.8)
Participants by risk factors ^a^			
No risk factors	285 (64.9)	96 (64.9)	381 (64.9)
with ≥1 risk factors	154 (35.1)	52 (35.1)	206 (35.1)

Data are for participants enrolled at Center for Indigenous Health sites. ^a^ Pre-defined risk factors include diabetes mellitus, chronic lung, liver, and heart disease, alcohol consumption, and tobacco use. Abbreviations: PCV13, 13-valent pneumococcal conjugate vaccine; V114, 15-valent pneumococcal conjugate vaccine.

**Table 2:** Summary of AEs Following Vaccination With V114/PCV13 and PPSV23.

n (%)	Following Vaccination with V114/PCV13 (Day 1–Month 6)		Following Vaccination with PPSV23 (Month 6–Month 7)	
	V114 n = 439	PCV13 n = 148	V114 n = 398	PCV13 n = 132
**Unsolicited or solicited AEs**				
**Any AE**	**356 (81.1)**	**114 (77.0)**	**250 (62.8)**	**85 (64.4)**
Injection-site ^a^	324 (73.8)	98 (66.2)	225 (56.5)	78 (59.1)
Systemic	246 (56.0)	91 (61.5)	157 (39.4)	54 (40.9)
**Any vaccine-related AE ^b^**	**335 (76.3)**	**105 (70.9)**	**237 (59.5)**	**80 (60.6)**
Systemic	193 (44.0)	65 (43.9)	125 (31.4)	45 (34.1)
**Any SAE**	**25 (5.7)**	**8 (5.4)**	**1 (0.3)**	**1 (0.8)**
**Any vaccine-related SAE ^b^**	**0 (0.0)**	**0 (0.0)**	**0 (0.0)**	**0 (0.0)**
**Deaths**	**2 (0.5)**	**1 (0.7)**	**0 (0.0)**	**0 (0.0)**
**Solicited AEs**				
**Solicited injection-site AEs ^c^**	**323 (73.6)**	**98 (66.2)**	**225 (56.5)**	**78 (59.1)**
Injection-site pain	316 (72.0)	96 (64.9)	219 (55.0)	74 (56.1)
Injection-site swelling	102 (23.2)	31 (20.9)	71 (17.8)	25 (18.9)
Injection-site erythema	60 (13.7)	17 (11.5)	53 (13.3)	22 (16.7)
**Solicited systemic AEs ^d^**	**212 (48.3)**	**77 (52.0)**	**141 (35.4)**	**50 (37.9)**
Fatigue	146 (33.3)	56 (37.8)	99 (24.9)	31 (23.5)
Headache	105 (23.9)	40 (27.0)	68 (17.1)	26 (19.7)
Myalgia	111 (25.3)	38 (25.7)	56 (14.1)	20 (15.2)
Arthralgia	69 (15.7)	20 (13.5)	44 (11.1)	17 (12.9)

Data are for participants enrolled at Center for Indigenous Health sites. Non-serious AEs reported between Days 1–14 following vaccination and SAEs reported between Day 1–Month 6 are included. ^a^ All injection-site AEs were considered vaccine-related by the investigator. ^b^ Relatedness determined by the investigator. ^c^ Solicited injection-site events were collected from Days 1–5 following vaccination. ^d^ Solicited systemic events were collected from Days 1–14 following vaccination. Abbreviations: AE, adverse event; PCV13, 13-valent pneumococcal conjugate vaccine; PPSV23, 23-valent pneumococcal polysaccharide vaccine; SAE, serious adverse event; V114, 15-valent pneumococcal conjugate vaccine.

**Table 3:** Pneumococcal NP/OP Carriage Prevalence by PCR in the Per-Protocol Population.

Pneumococcal Carriage Prevalence, % (n/N)			
Visit	V114	PCV13	Total
Day 1	23.9 (54/226)	18.7 (14/75)	22.6 (68/301)
Day 3	22.4 (47/210)	15.5 (11/71)	20.6 (58/281)
Day 8	20.7 (42/203)	16.7 (11/66)	19.7 (53/269)
Day 15	20.2 (42/208)	9.0 (6/67)	17.5 (48/275)
Day 30	18.6 (41/220)	10.3 (7/68)	16.7 (48/288)
Month 6	18.6 (37/199)	20.3 (13/64)	19.0 (50/263)
Day 183	18.0 (34/189)	15.3 (9/59)	17.3 (43/248)
Day 188	17.8 (33/185)	19.3 (11/57)	18.2 (44/242)
Day 195	20.4 (39/191)	10.9 (6/55)	18.3 (45/246)
Month 7	20.7 (42/203)	11.3 (7/62)	18.5 (49/265)

Data are for participants enrolled at Center for Indigenous Health sites. Carriage defined as detection of at least two pneumococcal genes by PCR: *lytA* and *cpsA* and/or *piaA*. N is the number of participants randomized and vaccinated in the substudy; n is the number of participants contributing to the analysis. Abbreviations: NP, nasopharyngeal; OP, oropharyngeal; PCV13, 13-valent pneumococcal conjugate vaccine; V114, 15-valent pneumococcal conjugate vaccine.

## Data Availability

The data sharing policy, including restrictions, of Merck Sharp & Dohme LLC, a subsidiary of Merck & Co., Inc., Rahway, NJ, USA (MSD), is available at https://engagezone.msd.com. Requests for access to the clinical study data can be submitted through the Engage Zone site or via email to dataaccess@msd.com. The data for this sub-analysis belong to the participating tribes. Data may be made available on request (contact lhammitt@jhu.edu), if use of data is consistent with the institutional review board-approved protocol and is approved by the participating tribes.

## Supplementary Materials

The following supporting information can be downloaded at https://www.mdpi.com/article/10.3390/vaccines14010003/s1. Table S1. List of investigators at CIH sites; Table S2. Subgroup analysis inclusion and exclusion criteria; Table S3. Summary of AEs following vaccination with V114 or PCV13 by age group (Day 1–Month 6); Table S4. Summary of AEs following vaccination with V114 or PCV13 by number of baseline risk factors (Day 1–Month 6); Table S5. Summary of OPA GMTs; Table S6. Summary of IgG GMCs; Table S7. Summary of OPA GMTs at 30 days following vaccination with V114 or PCV13 in participants 18–29 years of age; Table S8. Summary of OPA GMTs at 30 days following vaccination with V114 or PCV13 in participants 30–39 years of age; Table S9. Summary of OPA GMTs at 30 days following vaccination with V114 or PCV13 in participants 40–49 years of age; Table S10. Summary of OPA GMTs at 30 days following vaccination with V114 or PCV13; Table S11. Pneumococcal NP/OP carriage prevalence for V114 vaccine serotypes in the per-protocol population; Table S12. Pneumococcal NP/OP carriage prevalence for V114 vaccine serotypes in the per-protocol population; Figure S1. Proportion of participants with solicited AEs following vaccination with PPSV23.

## Abbreviations

The following abbreviations are used in this manuscript:

ACIP	Advisory Committee on Immunization Practices
AE	adverse event
AUDIT-C	Alcohol Use Disorder Identification Test Alcohol Consumption
CIH	Johns Hopkins Center for Indigenous Health
*cpsA*	capsular polysaccharide synthesis gene A
GMC	geometric mean concentrations
GMT	geometric mean titers
IgG	immunoglobulin G
IPD	invasive pneumococcal disease
*lytA*	autolysin
MSD	Merck Sharp & Dohme LLC, a subsidiary of Merck & Co., Inc., Rahway, NJ, USA
NP	nasopharyngeal
OP	oropharyngeal
OPA	opsonophagocytic activity
PCR	polymerase chain reaction
PCV	pneumococcal conjugate vaccine
PCV13	13-valent pneumococcal conjugate vaccine
PCV20	20-valent pneumococcal conjugate vaccine
PD	pneumococcal disease
*piaA*	pneumococcal iron acquisition
PPSV23	23-valent pneumococcal polysaccharide vaccine
SAE	serious adverse event
SSUAD	serotype-specific urinary antigen detection
V114	15-valent pneumococcal conjugate vaccine
