# Supplementary material for: Randomized Phase 3 Trial Evaluating the Safety, Tolerability, and Immunogenicity of V114, a 15-Valent PCV, Followed by PPSV23 6 Months Later (PNEU-DAY): Subgroup Analysis in Adults 18–49 Years of Age Enrolled at Center for Indigenous Health Sites
Source: Vaccines (Basel). 2025 Dec 19;14(1):3. doi: 10.3390/vaccines14010003 (PMC12846691; doi:10.3390/vaccines14010003)
Supplement: Supplementary file 1 [file vaccines-14-00003-s001.zip › vaccines-4026605-supplementary.pdf]

Table S1. List of investigators at CIH sites.

Facility Address	Primary Investigator	Sub-Investigators
Chinle Center for Indigenous Health<br>442 HIGHWAY 191<br>CHINLE, AZ 86503<br>USA	Laura Hammitt	Laura Brown, Lindsay Grant, Jane Halpern, Kirstin Howell, Jennifer Jaiyeola, Katherine OBrien, Alicia Portillo, Nina Ritchie, Kristen Roessler, Mathuram Santosham, Catherine Sutcliffe, Dan VanDeRiet, Bob Weatherholtz
Fort Defiance Center for Indigenous Health<br>1001 BONITO DRIVE<br>FORT DEFIANCE, AZ 86504<br>USA		Laura Brown, Estar Denny, Lindsay Grant, Jane Halpern, Kirstin Howell, Katherine OBrien, Alicia Portillo, Nina Ritchie, Kristen Roessler, Mathuram Santosham, Catherine Sutcliffe, Dan VanDeRiet, Bob Weatherholtz
Gallup Center for Indigenous Health<br>501 E. NIZHONI BLVD SUITE B<br>GALLUP, NM 87301<br>USA		Laura Brown, Lindsay Grant, Jane Halpern, Kirstin Howell, Katherine OBrien, Alicia Portillo, Nina Ritchie, Kristen Roessler, Mathuram Santosham, Catherine Sutcliffe, Carol Tso, Dan VanDeRiet, Bob Weatherholtz
Shiprock Center for Indigenous Health<br>1228 Yucca Dr.<br>SHIPROCK, NM 87420<br>USA		Laura Brown, Megan Gardner, Lindsay Grant, Jane Halpern, Kirstin Howell, Katherine OBrien, Alicia Portillo, Raymond Reid, Nina Ritchie, Kristen Roessler, Mathuram Santosham, Catherine Sutcliffe, Dan VanDeRiet, Bob Weatherholtz
Whiteriver Center for Indigenous Health<br>308 KUPER STREET<br>WHITERIVER, AZ 85941<br>USA		Laura Brown, Lindsay Grant, Jane Halpern, Kirstin Howell, Natalie Jones, Katherine OBrien, Alicia Portillo, Nina Ritchie, Kristen Roessler, Mathuram Santosham, Catherine Sutcliffe, Dan VanDeRiet, Bob Weatherholtz

Abbreviation: CIH, Center for Indigenous Health.

Table S2. Subgroup analysis inclusion and exclusion criteria.

**Inclusion Criteria**
1. Male or female 18–49 years of age (inclusive)
2. American Indian/Alaska Native from clinical sites of the CIH (formerly known as the CAIH):
A. In good health without any of the risk conditions for PD listed below (any underlying chronic illness must be documented to be in stable condition)
**OR**
B. With ≥1 of the following risk conditions for PD:
i. Diabetes mellitus type 1 or type 2, receiving treatment with ≥1 approved antidiabetic medication; HbA1c <10% at screening (Visit 1)
ii. Chronic liver disease with compensated cirrhosis (Child-Pugh Class A) due to non-alcoholic fatty liver disease, chronic hepatitis B, chronic hepatitis C, or alcoholic liver disease, diagnosed by clinician's assessment, with ≥1 of the following liver staging assessments (note: in cases in which >1 staging assessment is available, only one result may be used to determine fibrosis status. Biopsy should be preferred over every other test and FibroScan® over the blood test-based assessment. Participants with hepatitis C must have either completed a course of treatment with direct antiviral therapy and had a 12-week treatment-free follow-up or be expected to remain untreated during the duration of this study):
a. Prior liver biopsy demonstrating cirrhosis
b. FibroScan performed with an interpretable score >12.5 kPa within 5 years of Visit 1 (screening)
c. A FibroTest® (FibroSure®) with Fibrosis Score >0.75 performed within 5 years of Visit 1 and an APRI of >2 at Visit 1 (screening). APRI formula: AST ÷ laboratory upper limit of normal for AST × 100 ÷ (platelet count ÷ 100) (APRI calculation to be provided by the central laboratory)
d. Prior imaging study with evidence of cirrhosis and an APRI of >2 at Visit 1 (screening)
e. Prior imaging study with evidence of cirrhosis with splenomegaly and platelet count <120,000/μl at Visit 1 (screening)
iii. Confirmed diagnosis of COPD with spirometric data in the preceding 5 years showing post-bronchodilator FEV_1 over FVC ratio <0.7, and FEV_1 ≥30% predicted, corresponding to spirometric GOLD Stage 1 to 3 by severity assessment. For otherwise eligible individuals with a clinical history of COPD who do not have spirometry data from the preceding 5 years, spirometry can be performed at Visit 1 during the screening process by adequately trained personnel according to accepted guidelines

iv. Confirmed diagnosis of mild or moderate persistent asthma with documented reversible airflow obstruction on spirometry consistent with guidelines for the diagnosis of asthma performed within 5 years prior to Visit 1, and receipt of guideline-directed therapy for mild-to-moderate asthma. For otherwise eligible individuals with a clinical history of asthma who are receiving guideline-directed therapy for mild-to-moderate asthma but do not have spirometry data from the previous 5 years, spirometry can be performed at Visit 1 during the screening process by adequately trained personnel according to accepted guidelines for assessing reversibility. Inhaled bronchodilator and/or inhaled steroid therapy should be withheld prior to spirometry for a 'wash-out', as per recommended guidelines

v. Confirmed diagnosis of chronic heart disease due to one of the following conditions documented within the past 5 years (note: participants must have NYHA heart failure Class 1 to 3 at Visit 1 [screening] and receive guideline-directed oral heart failure treatment):

a. Heart failure with reduced EF: TTE or TEE with left ventricular EF <40%

b. Heart failure with preserved EF as diagnosed by a cardiologist based on clinical signs and symptoms, a TTE/TEE with left ventricular EF ≥50%, and either 1) plasma BNP >100 pg/ml or NT-proBNP >300 pg/ml or 2) at least one prior hospitalization or emergency room visit for heart failure

c. Non-cyanotic congenital heart disease

vi. Current smoker who has smoked at least 100 cigarettes during lifetime, is currently smoking every day or most days of the week, and is not currently receiving smoking cessation therapy at Visit 1 (screening) or planning to receive therapy during the study

vii. Based on the known association of alcohol abuse/misuse with increased morbidity and mortality of PD [60–63], all participants completed an AUDIT-C at screening. An AUDIT-C score of ≥5 identified individuals with potentially harmful alcohol use and counted as an additional risk factor for stratification purposes [37]

3. Receiving stable medical management for the conditions listed in inclusion criteria 1a–f, if applicable, for at least 3 months, with no anticipated major change expected for the duration of the study

4. A female participant is eligible to participate if she is not pregnant, not breastfeeding, and ≥1 of the following conditions applies:

a. Not a FOCBP

OR

b. A FOCBP who agrees to use contraceptive methods during the treatment period and for at least 6 weeks after the last dose of study intervention

5. The participant provides written informed consent for the study. The participant may also provide consent for future biomedical research. However, the participant may participate in the main study without participating in future biomedical research. American Indian/Alaska Native participants enrolled via the CIH will not participate in future biomedical research

Exclusion Criteria
1. History of active hepatitis with elevation in pre-treatment aspartate transaminase or alanine transaminase values >5 times the upper limit of normal within 3 months of Visit 1 (screening)
2. History of diabetic ketoacidosis or >1 episode of severe, symptomatic hypoglycemia within 3 months of Visit 1 (screening)
3. Myocardial infarction, acute coronary syndrome, transient ischemic attack, or ischemic or hemorrhagic stroke within 3 months of Visit 1 (screening)
4. History of severe pulmonary hypertension with WHO functional class ≥3, or history of Eisenmenger syndrome
5. History of invasive PD (positive blood culture, positive cerebrospinal fluid culture, or positive culture at another sterile site) or known history of other culture-positive PD within 3 years of Visit 2 (Day 1)
6. Known hypersensitivity to any component of pneumococcal polysaccharide vaccine, pneumococcal conjugate vaccine, or any diphtheria toxoid-containing vaccine
7. Known or suspected impairment of immunological function, including but not limited to a history of congenital or acquired immunodeficiency, documented HIV infection, functional or anatomic asplenia, or history of autoimmune disease
8. History of malignancy ≤5 years prior to signing informed consent (including hepatocellular carcinoma), except for adequately treated basal cell or squamous cell skin cancer or *in situ* cervical cancer
9. History of Stage 4 or 5 chronic kidney disease (glomerular filtration rate <30 ml/min/1.73 m^2) or nephrotic syndrome
10. History of alcohol withdrawal or alcohol withdrawal seizure in the past 12 months
11. History of coagulation disorder contraindicating intramuscular vaccinations
12. Recent febrile illness (defined as oral or tympanic temperature ≥100.4 °F [≥38.0 °C]; axillary or temporal temperature ≥99.4 °F [≥37.4 °C]; or rectal temperature ≥101.4 °F [≥38.6 °C]) or received antibiotic therapy for any acute illness occurring within 72 hours before receipt of study vaccine
13. History of hospitalization within 3 months of Visit 1 (screening)
14. Planned organ transplantation (heart, liver, lung, kidney, or pancreas) or other planned major surgery during the duration of this study
15. Expected survival for less than 1 year, according to the investigator’s judgment
16. A FOCBP who has a positive urine or serum pregnancy test before the first vaccination at Visit 2 (Day 1)

17. Prior administration of any pneumococcal vaccine or expected to receive any pneumococcal vaccine during the study outside of the protocol

18. Received systemic corticosteroids (prednisone equivalent of ≥20 mg/day) for ≥14 consecutive days and has not completed intervention at least 30 days before study entry

19. Received systemic corticosteroids exceeding physiologic replacement doses (approximately 5 mg/day prednisone equivalent) within 14 days before vaccination (note: topical, ophthalmic, intra-articular or soft tissue [e.g., bursa, tendon steroid injections], and inhaled/nebulized steroids are permitted)

20. Receiving immunosuppressive therapy, including chemotherapeutic agents used to treat cancer or other conditions, and interventions associated with organ or bone marrow transplantation, or autoimmune disease

21. Receiving immunomodulatory therapy with biological agents, such as monoclonal antibodies, directed against interleukin or cytokine pathways that could potentially interfere with immunogenicity assessment

22. Received any licensed, non-live vaccine within the 14 days before receipt of any study vaccine or is scheduled to receive any licensed, non-live vaccine within 30 days following receipt of any study vaccine. Exception: inactivated influenza vaccine may be administered but must be given at least 7 days before receipt of any study vaccine or at least 15 days after receipt of any study vaccine[a]

23. Received any live vaccine within 30 days before receipt of any study vaccine or is scheduled to receive any live vaccine within 30 days following receipt of any study vaccine

24. Received a blood transfusion or blood products, including immunoglobulins, within the 6 months before receipt of study vaccine or is scheduled to receive a blood transfusion or blood product within 30 days of receipt of study vaccine. Autologous blood transfusions are not considered an exclusion criterion

25. Receiving chronic home oxygen therapy

26. Is currently participating in, or has participated in, an interventional clinical study with an investigational compound or device within 2 months of participating in this current study

27. Is, at the time of signing informed consent, a user of recreational or illicit drugs or has had a recent history (within the past year) of drug abuse or dependence, as assessed by the study investigator

28. Has history or current evidence of any condition, therapy, laboratory abnormality, or other circumstance that might expose the participant to risk by participating in the study, confound the results of the study, or interfere with the participant's participation for the full duration of the study

29. Has or has had an immediate family member (e.g., spouse, parent/legal guardian, sibling, or child) who is an investigational site or sponsor staff member directly involved with this study

30. Diabetes mellitus with HbA1c ≥10% at Visit 1 (screening)

31. Chronic liver disease with Child-Pugh Class B or C cirrhosis at Visit 1 (screening)

32. Chronic lung disease with chronic obstructive pulmonary disease GOLD Stage 4 or severe persistent asthma at Visit 1 (screening)

33. Chronic heart disease with NYHA heart failure Class 4 at Visit 1 (screening)

[a]If the participant meets these exclusion criteria at screening, the Day 1 visit may be rescheduled for a time when these criteria are not met.

Abbreviations: APRI, AST-to-platelet ratio index; AST, aspartate aminotransferase; AUDIT-C, Alcohol Use Disorder Identification Test – Consumption; BNP, brain natriuretic peptide; CAIH, Center for American Indian Health; CIH, Center for Indigenous Health; COPD, chronic obstructive pulmonary disease; EF, ejection fraction; FEV_1, forced expiratory volume in 1 s; FOCBP, female of childbearing potential; FVC, forced vital capacity; HbA1c, glycated hemoglobin; GOLD, Global Initiative for Chronic Obstructive Lung Disease; HIV, human immunodeficiency virus; NT-proBNP, N-terminal pro B-type natriuretic peptide; NYHA, New York Heart Association; PD, pneumococcal disease; TEE, transesophageal echocardiography; TTE, transthoracic echocardiography; WHO, World Health Organization.

Table S3. Proportion of participants with solicited AEs following vaccination with V114/PCV13 and PPSV23 by maximum intensity.

		Following Vaccination with V114/PCV13 (Day 1–Month 6)		Following Vaccination with PPSV23 (Month 6–Month 7)	
n (%)	Intensity Grade	V114 n = 439	PCV13 n = 148	V114 n = 398	PCV13 n = 132
**Any solicited AE**	**Total**	**335 (76.3)**	**106 (71.6)**	**240 (60.3)**	**80 (60.6)**
	Mild	231 (52.6)	77 (52.0)	164 (41.2)	51 (38.6)
	Moderate	95 (21.6)	21 (14.2)	58 (14.6)	22 (16.7)
	Severe	9 (2.1)	8 (5.4)	18 (4.5)	7 (5.3)
**Solicited injection-site AEs**					
Injection-site pain	Total	316 (72.0)	96 (64.9)	219 (55.0)	74 (56.1)
	Mild	237 (54.0)	78 (52.7)	154 (38.7)	48 (36.4)
	Moderate	75 (17.1)	14 (9.5)	53 (13.3)	21 (15.9)
	Severe	4 (0.9)	4 (2.7)	12 (3.0)	5 (3.8)
Injection-site swelling[a]	Total	102 (23.2)	31 (20.9)	71 (17.8)	25 (18.9)
	Mild	80 (18.2)	29 (19.6)	49 (12.3)	16 (12.1)
	Moderate	20 (4.5)	1 (0.7)	20 (5.0)	6 (4.6)
	Severe	2 (0.5)	1 (0.7)	2 (0.5)	2 (1.5)
	Unknown	–	–	0	1 (0.8)
Injection-site erythema[a]	Total	60 (13.7)	17 (11.5)	53 (13.3)	22 (16.7)
	Mild	51 (11.6)	17 (11.5)	45 (11.3)	16 (12.1)
	Moderate	9 (2.1)	0	8 (2.1)	5 (3.8)
	Severe	0	0	0	0
	Unknown	–	–	0	1 (0.8)
**Solicited systemic AEs**					
Fatigue	Total	146 (33.3)	56 (37.8)	99 (24.9)	31 (23.5)
	Mild	115 (26.2)	45 (30.4)	75 (18.8)	25 (18.9)
	Moderate	26 (5.9)	9 (6.1)	20 (5.0)	6 (4.5)
	Severe	5 (1.1)	2 (1.4)	4 (1.0)	0
Myalgia	Total	111 (25.3)	38 (25.7)	56 (14.1)	20 (15.2)
	Mild	72 (16.4)	28 (18.9)	35 (8.8)	13 (9.8)
	Moderate	37 (8.4)	9 (6.1)	17 (4.3)	6 (4.5)
	Severe	2 (0.5)	1 (0.7)	4 (1.0)	1 (0.8)

Headache	Total	105 (23.9)	40 (27.0)	68 (17.1)	26 (19.7)
	Mild	83 (18.9)	30 (20.3)	52 (13.1)	21 (15.9)
	Moderate	21 (4.8)	9 (6.1)	14 (3.5)	4 (3.0)
	Severe	1 (0.2)	1 (0.7)	2 (0.5)	1 (0.8)
Arthralgia	Total	69 (15.7)	20 (13.5)	44 (11.1)	17 (12.9)
	Mild	53 (12.1)	19 (12.8)	27 (6.8)	8 (6.1)
	Moderate	15 (3.4)	1 (0.7)	13 (3.3)	9 (6.8)
	Severe	1 (0.2)	0	4 (1.0)	0

Data are for participants enrolled at Center for Indigenous Health sites. Solicited AEs were collected post-vaccination with PCV or PPSV23. Injection-site events were solicited from Days 1–5 following vaccination. Systemic events were solicited from Days 1–14 following vaccination.

[a]For solicited injection-site erythema and injection-site swelling, mild events measured >0–≤5 cm, moderate events measured >5–≤10 cm, and severe events measured >10 cm.

Abbreviations: AE, adverse event; PCV, pneumococcal conjugate vaccine; PCV13, 13-valent pneumococcal conjugate vaccine; PPSV23, 23-valent pneumococcal polysaccharide vaccine; V114, 15-valent pneumococcal conjugate vaccine.

Table S4. Summary of AEs following vaccination with V114 or PCV13 by age group (Day 1–Month 6).

	18–29 Years		30–39 Years		40–49 Years	
**n (%)**	**V114 n = 179**	**PCV13 n = 64**	**V114 n = 161**	**PCV13 n = 48**	**V114 n = 99**	**PCV13 n = 36**
Any AE	144 (80.4)	48 (75.0)	129 (80.1)	36 (75.0)	83 (83.8)	30 (83.3)
Injection-site[a]	135 (75.4)	43 (67.2)	112 (69.6)	32 (66.7)	77 (77.8)	23 (63.9)
Systemic	102 (57.0)	38 (59.4)	91 (56.5)	31 (64.6)	53 (53.5)	22 (61.1)
Any vaccine-related AE[b]	140 (78.2)	45 (70.3)	116 (72.0)	32 (66.7)	79 (79.8)	28 (77.8)
Systemic	83 (46.4)	26 (40.6)	73 (45.3)	22 (45.8)	37 (37.4)	17 (47.2)
Any SAE	6 (3.4)	4 (6.3)	12 (7.5)	4 (8.3)	7 (7.1)	0 (0.0)
Any vaccine-related SAE[a]	0 (0.0)	0 (0.0)	0 (0.0)	0 (0.0)	0 (0.0)	0 (0.0)
Deaths	1 (0.6)	1 (1.6)	0 (0.0)	0 (0.0)	1 (1.0)	0 (0.0)
Solicited AEs	142 (79.3)	46 (71.9)	118 (73.3)	33 (68.8)	80 (80.8)	28 (77.8)
Solicited injection-site AEs	135 (75.4)	43 (67.2)	111 (68.9)	32 (66.7)	77 (77.8)	23 (63.9)
Injection-site erythema	30 (16.8)	8 (12.5)	19 (11.8)	8 (16.7)	11 (11.1)	1 (2.8)
Injection-site pain	130 (72.6)	42 (65.6)	110 (68.3)	31 (64.6)	76 (76.8)	23 (63.9)
Injection-site swelling	50 (27.9)	11 (17.2)	35 (21.7)	12 (25.0)	17 (17.2)	8 (22.2)
Solicited systemic AEs	96 (53.6)	30 (50.0)	71 (44.1)	26 (54.2)	45 (45.5)	19 (52.8)
Arthralgia	23 (12.8)	5 (7.8)	29 (18.0)	10 (20.8)	17 (17.2)	5 (13.9)
Fatigue	64 (35.8)	21 (32.8)	51 (31.7)	22 (45.8)	31 (31.3)	13 (36.1)
Headache	49 (27.4)	14 (21.9)	34 (21.1)	14 (29.2)	22 (22.2)	12 (33.3)
Myalgia	45 (25.1)	18 (28.1)	41 (25.5)	13 (27.1)	25 (25.3)	7 (19.4)

[a]All injection-site AEs were considered vaccine-related by the investigator.
[b]Determined by the investigator to be related to the vaccine.
Abbreviations: AE, adverse event; PCV13, 13-valent pneumococcal conjugate vaccine; SAE, serious adverse event; V114, 15-valent pneumococcal conjugate vaccine.

Table S5. Summary of AEs following vaccination with V114 or PCV13 by number of baseline risk factors (Day 1–Month 6).

	No Risk Factors		≥1 Risk Factors	
n (%)	V114<br>n = 285	PCV13<br>n = 96	V114<br>n = 154	PCV13<br>n = 52
Any AE	234 (82.1)	71 (74.0)	122 (79.2)	43 (82.7)
Injection-site[a]	216 (75.8)	62 (64.6)	108 (70.1)	36 (69.2)
Systemic	167 (58.6)	55 (57.3)	79 (51.3)	36 (69.2)
Any vaccine-related AE[b]	221 (77.5)	65 (67.7)	114 (74.0)	40 (76.9)
Systemic	132 (46.3)	41 (42.7)	61 (39.6)	24 (46.2)
Any SAE	14 (4.9)	4 (4.2)	11 (7.1)	4 (7.7)
Any vaccine-related SAE[a]	0 (0.0)	0 (0.0)	0 (0.0)	0 (0.0)
Deaths	0 (0.0)	1 (1.0)	2 (1.3)	0 (0.0)
Solicited AEs	225 (78.9)	67 (69.8)	115 (74.7)	40 (76.9)
Solicited injection-site AEs	215 (75.4)	62 (64.6)	108 (70.1)	36 (69.2)
Injection-site erythema	40 (14.0)	12 (12.5)	20 (13.0)	5 (9.6)
Injection-site pain	208 (73.0)	60 (62.5)	108 (70.1)	36 (69.2)
Injection-site swelling	63 (22.1)	21 (21.9)	39 (25.3)	10 (19.2)
Solicited systemic AEs	145 (50.9)	48 (50.0)	67 (43.5)	29 (55.8)
Arthralgia	46 (16.1)	10 (10.4)	23 (14.9)	10 (19.2)
Fatigue	104 (36.5)	35 (36.5)	42 (27.3)	21 (40.4)
Headache	78 (27.4)	27 (28.1)	27 (17.5)	13 (25.0)
Myalgia	86 (30.2)	22 (22.9)	25 (16.2)	16 (30.8)

[a]All injection-site AEs were considered vaccine-related by the investigator.
[b]Determined by the investigator to be related to the vaccine.
Abbreviations: AE, adverse event; PCV13, 13-valent pneumococcal conjugate vaccine; SAE, serious adverse event; V114, 15-valent pneumococcal conjugate vaccine.

Table S6. Summary of OPA GMTs.

Pneumococcal Serotype	Timepoint	V114 (N = 439) n	V114 (N = 439) Observed GMT (95% CI)[a]	PCV13 (N = 148) n	PCV13 (N = 148) Observed GMT (95% CI)[a]
**13 shared serotypes**					
**1**	Day 1	434	5.4 (5.1–5.7)	146	6.2 (5.5–7.0)
	Day 30	412	171.7 (147.1–200.5)	132	141.1 (100.5–198.1)
	Month 6	375	46.9 (40.1–54.8)	129	52.5 (38.8–70.9)
	Month 7	359	201.9 (174.1–234.2)	120	172.0 (129.6–228.4)
**3**	Day 1	433	22.9 (20.3–25.7)	144	20.7 (16.5–26.0)
	Day 30	406	176.6 (154.9–201.2)	132	144.3 (112.1–185.7)
	Month 6	369	90.2 (78.9–103.2)	129	73.4 (57.3–93.9)
	Month 7	356	199.6 (175.7–226.8)	119	194.7 (152.0–249.4)
**4**	Day 1	432	47.3 (40.9–54.8)	142	55.1 (42.7–71.0)
	Day 30	409	1,000.8 (879.1–1,139.5)	134	2,089.4 (1713.8–2,547.4)
	Month 6	374	466.7 (402.3–541.4)	130	1,034.1 (832.9–1,283.8)
	Month 7	357	1,625.4 (1,472.5–1,794.2)	120	2,177.9 (1,887.5–2,512.9)
**5**	Day 1	437	19.8 (18.2–21.6)	147	20.6 (17.7–24.1)
	Day 30	411	431.7 (364.9–510.6)	134	668.9 (504.3–887.2)
	Month 6	373	163.8 (139.0–193.0)	130	215.5 (161.7–287.2)
	Month 7	360	515.5 (445.1–596.9)	120	669.2 (529.1–846.2)
**6A**	Day 1	394	479.6 (418.9–549.2)	130	388.3 (308.6–488.6)
	Day 30	407	13,954.4 (12,221.8–15,932.7)	129	10,906.6 (8,697.1–13,677.4)

	Month 6	371	5,693.4 (5,038.4–6,433.5)	129	4,772.9 (3,941.3–5,779.9)
	Month 7	353	6,246.2 (5,530.0–7,055.1)	117	5,405.4 (4,344.4–6,725.5)
**6B**	Day 1	423	245.4 (201.7–298.5)	143	234.0 (167.4–327.1)
	Day 30	407	11,697.9 (10,510.5–13,019.4)	134	8,077.0 (6,337.4–10,294.0)
	Month 6	375	5,174.1 (4,633.3–5,778.0)	130	4,212.1 (3,431.7–5,170.0)
	Month 7	360	5,663.4 (5,096.7–6,293.1)	120	5,130.9 (4,282.8–6,146.9)
**7F**	Day 1	403	825.2 (689.4–987.7)	135	807.8 (605.1–1,078.4)
	Day 30	412	5,206.9 (4,727.5–5,735.0)	134	7,601.8 (6,445.6–8,965.4)
	Month 6	373	3,528.3 (3,207.5–3,881.2)	129	4,383.2 (3,856.1–4,982.4)
	Month 7	360	6,519.4 (5,915.5–7,185.0)	120	6,596.1 (5,697.6–7,636.3)
**9V**	Day 1	425	920.3 (813.9–1,040.6)	146	771.5 (616.2–965.9)
	Day 30	410	3,092.6 (2,794.6–3,422.4)	134	3,608.1 (3,042.4–4,279.0)
	Month 6	374	2,138.0 (1,947.4–2,347.4)	129	2,136.3 (1,770.9–2,577.0)
	Month 7	360	3,505.3 (3,154.9–3,894.6)	120	3,263.8 (2,699.9–3,945.5)
**14**	Day 1	423	612.5 (517.7–724.6)	146	625.3 (480.3–814.0)
	Day 30	410	6,518.6 (5,763.9–7,372.3)	134	6,193.4 (5,123.0–7,487.3)
	Month 6	374	3,450.5 (3,076.6–3,869.9)	130	3,977.6 (3,322.3–4,762.0)
	Month 7	360	7,111.9 (6,393.4–7,911.2)	120	6,322.0 (5,276.4–7,574.8)
**18C**	Day 1	431	198.5 (172.3–228.8)	144	239.3 (186.1–307.7)
	Day 30	412	5,609.0 (5,046.6–6,234.2)	134	3,256.0 (2,655.7–3,992.1)
	Month 6	375	2,356.6 (2,118.1–2,621.8)	130	1,524.7 (1,251.6–1,857.4)

	Month 7	359	3,323.6 (3,008.9–3,671.2)	119	2,566.2 (2,186.5–3,011.9)
**19A**	Day 1	426	441.6 (382.9–509.4)	144	462.9 (364.3–588.2)
	Day 30	409	5,255.7 (4,733.1–5,836.0)	134	5,964.5 (4,859.7–7,320.5)
	Month 6	372	2,468.8 (2,245.9–2,713.8)	130	2,453.1 (2,057.0–2,925.3)
	Month 7	357	3,938.9 (3,546.1–4,375.3)	120	3,785.5 (3,175.1–4,513.2)
**19F**	Day 1	432	384.7 (336.5–440.0)	145	456.9 (361.9–577.0)
	Day 30	411	3,404.5 (3,100.8–3,738.0)	134	3,696.2 (3,115.7–4,384.9)
	Month 6	375	1,662.4 (1,512.7–1,827.0)	130	1,864.0 (1,563.2–2,222.7)
	Month 7	360	3,173.5 (2,910.5–3,460.3)	120	2,960.7 (2,518.3–3,480.8)
**23F**	Day 1	411	175.0 (145.0–211.3)	138	143.2 (105.5–194.3)
	Day 30	409	5,157.2 (4,554.9–5,839.2)	131	4,519.9 (3,647.6–5,600.9)
	Month 6	375	2,389.5 (2,082.1–2,742.3)	129	2,260.7 (1,787.6–2,858.9)
	Month 7	360	3,363.5 (2,972.9–3,805.4)	120	3,186.0 (2,626.3–3,865.0)
**Two additional serotypes in V114**					
**22F**	Day 1	394	299.0 (235.9–379.1)	137	267.6 (175.4–408.3)
	Day 30	408	3,097.8 (2,780.9–3,450.7)	124	356.7 (233.7–544.2)
	Month 6	369	1,981.2 (1,776.0–2,210.0)	120	441.4 (289.0–674.3)
	Month 7	358	3,313.0 (2,971.7–3,693.5)	118	2,893.3 (2,171.8–3,854.5)
**33F**	Day 1	431	2,832.6 (2,521.6–3,182.0)	146	3,072.1 (2,487.6–3,794.0)
	Day 30	407	11,674.1 (10,434.7–13,060.6)	134	2,907.7 (2,308.9–3,661.8)
	Month 6	375	7,409.0 (6,716.2–8,173.2)	129	3,233.7 (2,640.6–3,960.0)
	Month 7	356	12,983.8 (11,541.8–14,605.9)	120	13,038.3 (10,240.3–16,600.8)

N is the number of participants randomized and vaccinated; n is the number of participants contributing to the analysis. Per protocol, Day 1 is pre-vaccination with PCV, Day 30 is 30 days following vaccination with PCV, Month 6 is 6 months following vaccination with PCV and pre-vaccination with PPSV23, and Month 7 is 30 days following vaccination with PPSV23.
[a]The within-group 95% Cls are obtained by exponentiating the Cls of the mean of the natural log values based on the t-distribution.
Abbreviations: CI, confidence interval; GMT, geometric mean titer (1/dilution); OPA, opsonophagocytic activity; PCV, pneumococcal conjugate vaccine; PCV13, 13-valent pneumococcal conjugate vaccine; PPSV23, 23-valent pneumococcal polysaccharide vaccine; V114, 15-valent pneumococcal conjugate vaccine.

Table S7. Summary of IgG GMCs.

		V114 (N = 439)		PCV13 (N = 148)	
**Pneumococcal Serotype**	**Timepoint**	**n**	**Observed GMC (95% CI)[a]**	**n**	**Observed GMC (95% CI)[a]**
**13 shared serotypes**					
**1**	Day 1	439	0.24 (0.21–0.26)	147	0.25 (0.21–0.30)
	Day 30	412	2.75 (2.43–3.10)	134	3.94 (3.19–4.87)
	Month 6	375	0.94 (0.82–1.06)	130	1.37 (1.10–1.71)
	Month 7	360	2.22 (2.01–2.46)	120	2.73 (2.30–3.26)
**3**	Day 1	439	0.17 (0.15–0.19)	147	0.17 (0.14–0.20)
	Day 30	410	0.61 (0.55–0.67)	134	0.56 (0.46–0.67)
	Month 6	375	0.30 (0.27–0.33)	130	0.28 (0.24–0.34)
	Month 7	360	0.52 (0.47–0.57)	120	0.51 (0.44–0.61)
**4**	Day 1	439	0.15 (0.14–0.16)	147	0.15 (0.13–0.17)
	Day 30	410	1.03 (0.90–1.18)	134	1.97 (1.58–2.46)
	Month 6	373	0.42 (0.37–0.47)	130	0.64 (0.53–0.79)
	Month 7	359	0.95 (0.85–1.05)	119	1.21 (1.01–1.46)
**5**	Day 1	439	0.69 (0.64–0.74)	147	0.76 (0.66–0.88)
	Day 30	412	2.71 (2.35–3.13)	133	3.84 (2.89–5.08)
	Month 6	375	1.43 (1.27–1.62)	130	1.93 (1.54–2.43)
	Month 7	360	2.68 (2.35–3.06)	120	3.27 (2.60–4.10)
**6A**	Day 1	439	0.28 (0.25–0.31)	147	0.32 (0.26–0.39)
	Day 30	412	11.45 (9.78–13.41)	134	12.22 (9.22–16.18)
	Month 6	375	3.23 (2.76–3.78)	130	3.77 (2.86–4.96)
	Month 7	360	3.28 (2.82–3.81)	120	4.18 (3.19–5.47)
**6B**	Day 1	439	0.41 (0.37–0.46)	147	0.41 (0.34–0.51)

	Day 30	412	20.41 (17.49–23.83)	133	14.61 (10.97–19.47)
	Month 6	375	5.55 (4.74–6.49)	130	4.49 (3.35–6.01)
	Month 7	360	5.82 (5.02–6.74)	120	5.95 (4.56–7.76)
**7F**	Day 1	439	0.36 (0.33–0.40)	147	0.34 (0.28–0.41)
	Day 30	412	4.15 (3.65–4.73)	134	5.03 (3.97–6.37)
	Month 6	375	1.48 (1.31–1.67)	130	1.64 (1.31–2.04)
	Month 7	360	2.58 (2.29–2.90)	120	2.64 (2.18–3.19)
**9V**	Day 1	439	0.39 (0.36–0.43)	147	0.40 (0.33–0.47)
	Day 30	411	3.82 (3.39–4.32)	134	5.05 (4.06–6.28)
	Month 6	374	1.58 (1.41–1.77)	130	1.92 (1.58–2.34)
	Month 7	360	2.54 (2.28–2.83)	120	2.85 (2.37–3.44)
**14**	Day 1	439	0.98 (0.85–1.12)	147	1.00 (0.78–1.27)
	Day 30	412	14.22 (12.22–16.53)	134	15.60 (12.28–19.82)
	Month 6	375	6.14 (5.33–7.07)	130	8.07 (6.49–10.05)
	Month 7	360	11.08 (9.86–12.45)	120	10.67 (8.74–13.03)
**18C**	Day 1	439	0.38 (0.35–0.42)	147	0.41 (0.34–0.49)
	Day 30	412	13.49 (11.79–15.44)	134	9.88 (7.76–12.58)
	Month 6	375	3.77 (3.31–4.31)	130	3.19 (2.54–4.00)
	Month 7	360	4.32 (3.82–4.89)	120	3.73 (3.03–4.61)
**19A**	Day 1	439	1.98 (1.82–2.15)	147	1.83 (1.55–2.16)
	Day 30	412	25.23 (22.53–28.26)	134	32.69 (26.54–40.25)
	Month 6	375	8.12 (7.29–9.04)	130	9.86 (8.07–12.04)
	Month 7	360	11.84 (10.66–13.15)	120	13.13 (10.93–15.77)
**19F**	Day 1	439	1.10 (0.98–1.23)	147	1.05 (0.86–1.28)

	Day 30	411	20.44 (18.22–22.94)	134	20.69 (16.83–25.44)
	Month 6	375	6.31 (5.61–7.10)	130	6.42 (5.22–7.89)
	Month 7	360	10.22 (9.21–11.35)	120	10.38 (8.68–12.43)
**23F**	Day 1	439	0.49 (0.44–0.54)	147	0.48 (0.40–0.57)
	Day 30	412	13.84 (12.11–15.81)	134	15.19 (12.01–19.21)
	Month 6	374	4.06 (3.57–4.61)	130	4.48 (3.57–5.63)
	Month 7	360	4.54 (4.02–5.11)	119	5.25 (4.27–6.44)
**Two additional serotypes in V114**					
**22F**	Day 1	439	0.85 (0.75–0.95)	147	0.75 (0.62–0.91)
	Day 30	412	6.26 (5.56–7.06)	134	0.75 (0.62–0.92)
	Month 6	375	2.67 (2.38–2.99)	130	0.70 (0.58–0.86)
	Month 7	360	4.71 (4.22–5.25)	120	3.71 (2.85–4.82)
**33F**	Day 1	439	0.98 (0.88–1.10)	147	0.96 (0.78–1.19)
	Day 30	412	7.60 (6.63–8.72)	133	0.95 (0.77–1.16)
	Month 6	375	3.21 (2.81–3.66)	130	0.88 (0.72–1.07)
	Month 7	360	5.57 (4.89–6.34)	120	7.56 (5.76–9.93)

N is the number of participants randomized and vaccinated; n is the number of participants contributing to the analysis. Per protocol, Day 1 is pre-vaccination with PCV, Day 30 is 30 days following vaccination with PCV, Month 6 is 6 months following vaccination with PCV and pre-vaccination with PPSV23, and Month 7 is 30 days following vaccination with PPSV23.

[a]The within-group 95% Cls are obtained by exponentiating the Cls of the mean of the natural log values based on the t-distribution.

CI = confidence interval; GMC = geometric mean concentration; IgG = immunoglobulin G; PCV = pneumococcal conjugate vaccine; PCV13 = 13-valent pneumococcal conjugate vaccine; PPSV23 = 23-valent pneumococcal polysaccharide vaccine; V114 = 15-valent pneumococcal conjugate vaccine.

Table S8. Summary of OPA GMTs at 30 days following vaccination with V114 or PCV13 in participants 18–29 years of age.

	V114 (N = 179)		PCV13 (N = 64)	
**Pneumococcal Serotype**	**n**	**Observed GMT (95% CI)[a]**	**n**	**Observed GMT (95% CI)[a]**
**13 shared serotypes**				
1	163	250.7 (199.6–314.9)	56	152.0 (88.7–260.4)
3	162	169.4 (141.5–202.8)	55	135.8 (93.8–196.5)
4	162	1,066.4 (882.9–1,288.0)	57	2,444.5 (1,880.3–3,177.9)
5	162	588.1 (469.4–736.7)	57	681.3 (454.8–1,020.7)
6A	159	18,533.9 (15,326.0–22,413.1)	55	16,013.8 (11,881.7–21,582.8)
6B	161	13,244.7 (11,257.4–15,582.9)	57	11,073.1 (7653.9–16,019.9)
7F	163	6,184.0 (5,325.9–7,180.3)	57	7,805.1 (5,988.6–10,172.7)
9V	162	3,761.0 (3,247.6–4,355.6)	57	4,767.4 (3,806.8–5,970.4)
14	162	9,087.3 (7,682.9–10,748.4)	57	9,211.4 (6,857.8–12,372.6)
18C	163	6,471.6 (5,611.6–7,463.4)	57	3,488.7 (2,566.4–4,742.5)
19A	162	5,558.9 (4,776.3–6,469.7)	57	6,323.2 (4,809.5–8,313.2)
19F	163	3,850.9 (3,309.2–4,481.2)	57	3,909.5 (2,996.0–5,101.4)
23F	161	6,263.7 (5,200.3–7,544.7)	57	5,856.0 (4,323.1–7,932.6)
**Two additional serotypes in V114**				
22F	163	3,643.0 (3,087.1–4,298.9)	52	387.3 (194.1–772.9)
33F	160	12,775.9 (10,687.0–15,273.2)	57	3,609.7 (2,605.2–5,001.5)

N is the number of participants randomized and vaccinated; n is the number of participants contributing to the analysis.

[a]The within-group 95% CIs are obtained by exponentiating the CIs of the mean of the natural log values based on the t-distribution.

Abbreviations: CI , confidence interval; GMT, geometric mean titer (1/dilution); OPA,

opsonophagocytic activity; PCV13, 13-valent pneumococcal conjugate vaccine; V114, 15-valent pneumococcal conjugate vaccine.

Table S9. Summary of OPA GMTs at 30 days following vaccination with V114 or PCV13 in participants 30–39 years of age.

	V114 (N = 161)		PCV13 (N = 48)	
**Pneumococcal Serotype**	**n**	**Observed GMT (95% CI)[a]**	**n**	**Observed GMT (95% CI)[a]**
**13 shared serotypes**				
1	152	160.2 (123.8–207.2)	45	185.1 (101.2–338.8)
3	151	169.2 (132.6–215.9)	46	171.3 (112.2–261.6)
4	151	1,106.0 (911.2–1,342.4)	46	2,134.5 (1,455.0–3,131.3)
5	152	384.5 (285.4–517.9)	46	725.9 (453.5–1,161.8)
6A	151	13,379.4 (10,570.7–16,934.2)	44	8,314.7 (5,450.8–12,683.2)
6B	149	12,575.5 (10,736.6–14,729.4)	46	7,582.5 (5,160.2–11,141.8)
7F	152	4,970.7 (4,327.3–5,709.8)	46	8,741.7 (6,709.8–11,388.8)
9V	151	3,003.1 (2,552.5–3,533.2)	46	3,110.4 (2,337.0–4,139.7)
14	151	5,684.8 (4,577.5–7,059.9)	46	5,028.0 (3,635.8–6,953.3)
18C	152	5,481.3 (4,502.1–6,673.6)	46	3,601.8 (2,571.7–5,044.4)
19A	152	5,673.6 (4,776.5–6,739.3)	46	6,722.0 (4,944.7–9,138.2)
19F	152	3,320.1 (2,882.9–3,823.7)	46	3,982.9 (3,117.7–5,088.2)
23F	151	5,116.9 (4,175.0–6,271.4)	44	4,943.8 (3,442.3–7,100.2)
**Two additional serotypes in V114**				
22F	152	2,907.0 (2,451.0–3,447.9)	44	370.8 (186.9–735.9)
33F	151	12,045.9 (10,078.6–14,397.0)	46	2,541.9 (1,714.3–3,769.0)

N is the number of participants randomized and vaccinated; n is the number of participants contributing to the analysis.

[a]The within-group 95% CIs are obtained by exponentiating the CIs of the mean of the natural log values based on the t-distribution.

CI = confidence interval; GMT = geometric mean titer (1/dilution); OPA = opsonophagocytic activity; PCV13 = 13-valent pneumococcal conjugate vaccine; V114 = 15-valent pneumococcal conjugate vaccine.

Table S10. Summary of OPA GMTs at 30 days following vaccination with V114 or PCV13 in participants 40–49 years of age.

	V114 (N = 99)		PCV13 (N = 36)	
**Pneumococcal Serotype**	**n**	**Observed GMT (95% CI)[a]**	**n**	**Observed GMT (95% CI)[a]**
**13 shared serotypes**				
1	97	101.4 (72.8–141.3)	31	83.1 (43.0–160.4)
3	93	203.2 (154.5–267.3)	31	124.5 (66.7–232.5)
4	96	768.5 (549.1–1,075.6)	31	1,516.9 (969.8–2,372.7)
5	97	308.8 (211.2–451.5)	31	572.7 (275.4–1,190.9)
6A	97	9,356.9 (7,165.3–12,218.9)	30	8,030.0 (4,842.4–13,315.9)
6B	97	8,517.7 (6,560.2–11,059.4)	31	4,966.1 (2,827.5–8,722.2)
7F	97	4,194.5 (3,302.7–5,327.2)	31	5,885.7 (4,091.1–8,467.5)
9V	97	2,334.9 (1,839.9–2,963.1)	31	2,694.5 (1,742.1–4,167.7)
14	97	4,631.6 (3,558.0–6,029.2)	31	4,067.0 (2,916.6–5,671.2)
18C	97	4,572.7 (3,662.4–5,709.3)	31	2,469.1 (1,515.0–4,024.1)
19A	95	4,226.0 (3,297.0–5,416.7)	31	4,486.4 (2,463.5–8,170.4)
19F	96	2,873.8 (2,328.2–3,547.3)	31	2,984.2 (1,912.2–4,657.3)
23F	97	3,781.0 (2,865.3–4,989.4)	30	2,422.9 (1,484.0–3,955.9)
**Two additional serotypes in V114**				
22F	93	2,586.8 (2,014.7–3,321.4)	28	287.9 (111.7–741.7)
33F	96	9,561.4 (7,470.2–12,238.2)	31	2,385.1 (1,342.1–4,238.8)

N is the number of participants randomized and vaccinated; n is the number of participants contributing to the analysis.

[a]The within-group 95% CIs are obtained by exponentiating the CIs of the mean of the natural log values based on the t-distribution.

CI = confidence interval; GMT = geometric mean titer (1/dilution); OPA = opsonophagocytic activity; PCV13 = 13-valent pneumococcal conjugate vaccine; V114 = 15-valent pneumococcal conjugate vaccine.

Table S11. Summary of OPA GMTs at 30 days following vaccination with V114 or PCV13.

A. Participants with no risk factors

	V114 (N = 285)		PCV13 (N = 96)	
**Pneumococcal Serotype**	**n**	**Observed GMT (95% CI)[a]**	**n**	**Observed GMT (95% CI)[a]**
**13 shared serotypes**				
1	266	166.9 (137.2–202.9)	87	122.7 (79.6–189.0)
3	263	160.2 (137.4–186.8)	87	155.0 (113.3–212.1)
4	265	1,066.5 (918.3–1,238.7)	88	2,196.6 (1,678.8–2,874.2)
5	265	456.8 (371.2–562.0)	88	695.3 (494.7–977.1)
6A	263	14,092.5 (11,948.5–16,621.2)	86	9,977.4 (7,437.1–13,385.6)
6B	265	11,847.9 (10,368.0–13,539.1)	88	8,406.3 (6,254.4–11,298.6)
7F	266	5,186.3 (4,651.9–5,782.1)	88	7,593.1 (6,184.8–9,322.1)
9V	264	3,013.3 (2,639.1–3,440.5)	88	3,657.5 (2,871.6–4,658.5)
14	266	6,300.5 (5,433.0–7,306.5)	88	6,061.1 (4,720.7–7,782.2)
18C	266	5,357.5 (4,692.4–6,116.9)	88	3,155.5 (2,494.2–3,992.0)
19A	263	5,362.3 (4,729.5–6,079.7)	88	5,453.4 (4,438.6–6,700.2)
19F	266	3,387.7 (3,007.3–3,816.3)	88	3,674.6 (2,993.7–4,510.4)
23F	265	5,076.5 (4,422.7–5,826.9)	87	4,901.8 (3,812.3–6,302.6)
**Two additional serotypes in V114**				
22F	265	3,140.6 (2,773.4–3,556.3)	84	549.7 (346.9–871.0)
33F	263	11,701.9 (10,227.7–13,388.6)	88	3,380.1 (2,550.1–4,480.2)

B. Participants with ≥1 risk factors

	V114 (N = 154)		PCV13 (N = 52)	
**Pneumococcal Serotype**	**n**	**Observed GMT (95% CI)[a]**	**n**	**Observed GMT (95% CI)[a]**
**13 shared serotypes**				
1	146	181.0 (140.1–233.8)	45	184.8 (106.0–322.3)

3	143	211.1 (166.1–268.3)	45	125.6 (81.0–195.0)
4	144	890.4 (696.0–1,138.9)	46	1,898.7 (1,444.1–2,496.5)
5	146	389.6 (291.9–520.1)	46	621.1 (368.8–1,046.0)
6A	144	13,705.8 (10,941.5–17,168.5)	43	13,032.5 (9181.2–18,499.5)
6B	142	11,422.9 (9,528.3–13,694.4)	46	7,482.4 (4,826.0–11,601.1)
7F	146	5,244.8 (4,341.2–6,336.6)	46	7,618.4 (5,709.7–10,165.0)
9V	146	3,241.5 (2,776.1–3,784.9)	46	3,515.6 (2,898.4–4,264.1)
14	144	6,941.7 (5,565.6–8,658.0)	46	6,454.4 (4,831.3–8,622.8)
18C	146	6,098.0 (5,112.8–7,273.0)	46	3,457.5 (2,316.9–5,159.6)
19A	146	5,069.0 (4,196.9–6,122.4)	46	7,079.6 (4,479.1–11,190.1)
19F	145	3,435.5 (2,952.4–3,997.7)	46	3,737.9 (2,720.0–5,136.9)
23F	144	5,309.2 (4,145.2–6,800.1)	44	3,850.3 (2,551.8–5,809.6)
**Two additional serotypes in V114**				
22F	143	3,020.0 (2,457.0–3,712.0)	40	143.8 (61.7–335.1)
33F	144	11,623.4 (9,491.6–14,234.0)	46	2,180.0 (1,458.1–3,259.4)

N is the number of participants randomized and vaccinated; n is the number of participants contributing to the analysis.

[a]The within-group 95% CIs are obtained by exponentiating the CIs of the mean of the natural log values based on the t-distribution.

Abbreviations: CI = confidence interval; GMT = geometric mean titer (1/dilution); OPA = opsonophagocytic activity; PCV13 = 13-valent pneumococcal conjugate vaccine; V114 = 15-valent pneumococcal conjugate vaccine.

Table S12. Pneumococcal NP/OP carriage prevalence for V114 vaccine serotypes in the per-protocol population.

Serotype	Timepoint	V114, % (n/N) N = 226	PCV13, % (n/N) N = 75	Total, % (n/N) N = 301
1	Day 1	4.0 (9/226)	2.7 (2/75)	3.7 (11/301)
	Month 6	1.5 (3/199)	1.6 (1/64)	1.5 (4/263)
	Month 7	3.0 (6/203)	0.0 (0/62)	2.3 (6/265)
3	Day 1	2.2 (5/226)	2.7 (2/75)	2.3 (7/301)
	Month 6	1.5 (3/199)	1.6 (1/64)	1.5 (4/263)
	Month 7	2.0 (4/203)	0.0 (0/62)	1.5 (4/265)
4	Day 1	6.6 (15/226)	4.0 (3/75)	6.0 (18/301)
	Month 6	5.5 (11/199)	6.3 (4/64)	5.7 (15/263)
	Month 7	6.4 (13/203)	1.6 (1/62)	5.3 (14/265)
5	Day 1	4.9 (11/226)	5.3 (4/75)	5.0 (15/301)
	Month 6	1.5 (3/199)	1.6 (1/64)	1.5 (4/263)
	Month 7	3.0 (6/203)	1.6 (1/62)	2.6 (7/265)
6A	Day 1	0.0 (0/226)	0.0 (0/75)	0.0 (0/301)
	Month 6	0.0 (0/199)	0.0 (0/64)	0.0 (0/263)
	Month 7	0.5 (1/203)	0.0 (0/62)	0.4 (1/265)
6B	Day 1	0.0 (0/226)	0.0 (0/75)	0.0 (0/301)
	Month 6	0.0 (0/199)	0.0 (0/64)	0.0 (0/263)
	Month 7	0.5 (1/203)	0.0 (0/62)	0.4 (1/265)
7F	Day 1	0.9 (2/226)	1.3 (1/75)	1.0 (3/301)
	Month 6	0.0 (0/199)	1.6 (1/64)	0.4 (1/263)
	Month 7	2.0 (4/203)	0.0 (0/62)	1.5 (4/265)
9V	Day 1	5.8 (13/226)	9.3 (7/75)	6.6 (20/301)
	Month 6	4.5 (9/199)	4.7 (3/64)	4.6 (12/263)
	Month 7	3.4 (7/203)	4.8 (3/62)	3.8 (10/265)
14	Day 1	0.4 (1/226)	0.0 (0/75)	0.3 (1/301)
	Month 6	0.0 (0/199)	1.6 (1/64)	0.4 (1/263)
	Month 7	0.0 (0/203)	0.0 (0/62)	0.0 (0/265)
18C	Day 1	0.4 (1/226)	0.0 (0/75)	0.3 (1/301)
	Month 6	1.0 (2/199)	0.0 (0/64)	0.8 (2/263)
	Month 7	1.5 (3/203)	0.0 (0/62)	1.1 (3/265)
19A	Day 1	0.0 (0/226)	2.7 (2/75)	0.7 (2/301)
	Month 6	0.0 (0/199)	1.6 (1/64)	0.4 (1/263)
	Month 7	0.0 (0/203)	1.6 (1/62)	0.4 (1/265)
19F	Day 1	2.7 (6/226)	0.0 (0/75)	2.0 (6/301)
	Month 6	1.0 (2/199)	1.6 (1/64)	1.1 (3/263)
	Month 7	0.5 (1/203)	0.0 (0/62)	0.4 (1/265)
23F	Day 1	0.4 (1/226)	0.0 (0/75)	0.3 (1/301)

	Month 6	0.0 (0/199)	0.0 (0/64)	0.0 (0/263)
	Month 7	0.0 (0/203)	0.0 (0/62)	0.0 (0/265)
**Two additional serotypes in V114**				
22F	Day 1	3.1 (7/226)	1.3 (1/75)	2.7 (8/301)
	Month 6	1.5 (3/199)	1.6 (1/64)	1.5 (4/263)
	Month 7	1.0 (2/203)	1.6 (1/62)	1.1 (3/265)
33F	Day 1	5.8 (13/226)	6.7 (5/75)	6.0 (18/301)
	Month 6	4.0 (8/199)	7.8 (5/64)	4.9 (13/263)
	Month 7	3.4 (7/203)	6.5 (4/62)	4.2 (11/265)

Specimens confirmed as positive for pneumococcal carriage via detection of at least two pneumococcal genes (*lytA,* and *cpsA* and/or *piaA*) were subjected to triplex real-time multiplex PCRs spanning the 15 serotypes covered in V114.
N is the number of participants randomized and vaccinated in the substudy; n is the number of participants contributing to the analysis. Percentage is calculated as 100 × number of participants in applicable category/number of participants with non-missing results for each sample type in the per-protocol population per serotype at each timepoint. Per protocol, Day 1 is pre-vaccination with PCV and Month 6 is 6 months following vaccination with PCV and pre-vaccination with PPSV23.
Abbreviations: NP, nasopharyngeal; OP, oropharyngeal; PCR, polymerase chain reaction; PCV, pneumococcal conjugate vaccine; PCV13, 13-valent pneumococcal conjugate vaccine; PPSV23, 23-valent pneumococcal polysaccharide vaccine; V114,15-valent pneumococcal conjugate vaccine.

Figure S1. Proportion of participants with solicited AEs following vaccination with PPSV23.

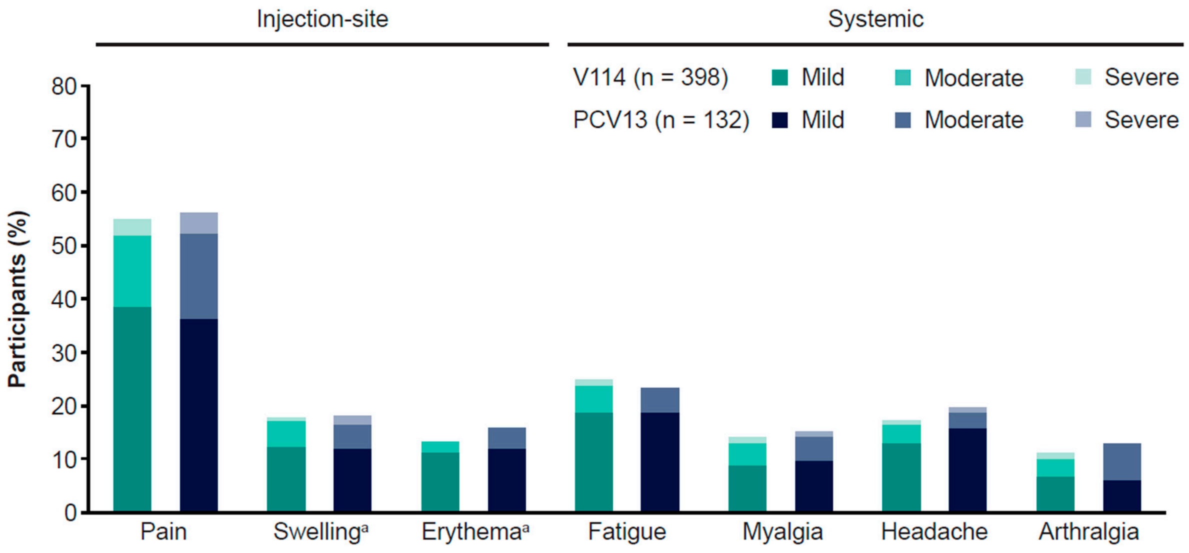

Injection-site events were solicited from Days 1–5 following vaccination. Systemic events were solicited from Days 1–14 following vaccination.
[a]For solicited injection-site erythema and injection-site swelling, mild events measured >0–≤5 cm, moderate events measured >5–≤10 cm, and severe events measured >10 cm. The maximum size was unknown for one participant with injection-site erythema and injection-site swelling in the PCV13 group.
Abbreviations: AE, adverse event; PCV13, 13-valent pneumococcal conjugate vaccine; PPSV23, 23-valent pneumococcal polysaccharide vaccine; V114, 15-valent pneumococcal conjugate vaccine.

References

37. Government of Canada. CAPVAXIVE. Available online: https://pdf.hres.ca/dpd_pm/00076331.PDF (accessed on 1 January 2025).
60. Farrar, J.L.; Childs, L.; Ouattara, M.; Akhter, F.; Britton, A.; Pilishvili, T.; Kobayashi, M. Systematic review and meta-analysis of the efficacy and effectiveness of pneumococcal vaccines in adults. *Pathogens* **2023**, *12*, 732. https://doi.org/10.3390/pathogens12050732.
61. Arya, S.; Norton, N.; Kaushik, P.; Brandtmuller, A.; Tsoumani, E. Recent changes to adult national immunization programs for pneumococcal vaccination in Europe and how they impact coverage: A systematic review of published and grey literature. *Hum. Vaccin. Immunother.* **2023**, *19*, 2279394. https://doi.org/10.1080/21645515.2023.2279394.
62. Ngamprasertchai, T.; Ruenroengbun, N.; Kajeekul, R. Immunogenicity and safety of the higher-valent pneumococcal conjugate vaccine vs the 13-valent pneumococcal conjugate vaccine in older adults: A systematic review and meta-analysis of randomized controlled trials. *Open Forum Infect. Dis.* **2025**, *12*, ofaf069. https://doi.org/10.1093/ofid/ofaf069.
63. Schellenberg, J.J.; Adam, H.J.; Baxter, M.R.; Karlowsky, J.A.; Golden, A.R.; Martin, I.; Demczuk, W.; Mulvey, M.R.; Zhanel, G.G. Comparison of PCV10, PCV13, PCV15, PCV20 and PPSV23 vaccine coverage of invasive Streptococcus pneumoniae isolate serotypes in Canada: The SAVE study, 2011–2020. *J. Antimicrob. Chemother.* **2023**, *78*, i37–i47. https://doi.org/10.1093/jac/dkad068.
